# Discordance between reported discrimination and translational-readiness evidence in AI-based ASCVD prediction: a systematic review with structured methodological audit

**DOI:** 10.3389/fpubh.2026.1963871

**Published:** 2026-09-30

**Authors:** Ruifeng Liu, Ross Arena, Carl Lavie, Adelaide M. Arruda-Olson, Dejana Popovic

**Affiliations:** 1 Beijing Friendship Hospital, Capital Medical University, Beijing, China; 2 Department of Physical Therapy, University of Illinois at Chicago, Chicago, IL, United States; 3 HealthPartners Institute, Minneapolis, MN, United States; 4 John L Ochsner Heart and Vascular Institute, New Orleans, LA, United States; 5 Mayo Clinic, Rochester, MN, United States

**Keywords:** artificial intelligence, clinical utility, machine learning, methodological audit, reproducibility, risk prediction models, translational evidence gaps

## Abstract

**Importance:**

The development of artificial intelligence (AI) models for atherosclerotic cardiovascular disease (ASCVD) risk prediction has rapidly accelerated. However, despite reports of high predictive accuracy, evidence supporting clinical implementation remains limited.

**Objective:**

This study aimed to evaluate the methodological rigor of AI-based ASCVD prediction models and assess whether reported algorithmic discrimination is associated with prespecified reporting and translational-evidence gaps.

**Data sources and study selection:**

A systematic search of PubMed, Embase, Web of Science, Scopus, and Cochrane CENTRAL was conducted for original studies published between 1 January 2020 and 27 March 2026. Studies reporting the development or validation of AI and machine learning prognostic models for adult ASCVD outcomes were included.

**Data extraction and synthesis:**

Following the PRISMA guidelines, a structured methodological audit was performed. Five binary reporting and translational-evidence gaps were independently extracted: absent calibration assessment, lack of external validation, absent decision-analytic utility evaluation, undefined temporal prediction horizons, and lack of reproducibility or accessibility artifacts. A prespecified minimum evidence proxy for translational evaluation was defined.

**Main outcomes and measures:**

The primary outcomes were the cumulative methodological gap burden (range, 0–5) and its association with the primary discrimination metric (area under the receiver operating characteristic curve [AUC] or C-index).

**Results:**

Among 101 primary models evaluated, the median discrimination performance was 0.790 (IQR, 0.734–0.833). However, a substantial cumulative gap burden was observed, with only 6.9% of the models meeting the prespecified translational-readiness proxy. Reported discrimination was not associated with methodological completeness; top-quartile models (AUC ≥ 0.833) exhibited high gap burdens at rates comparable to lower-performing models. Reporting and translational-evidence gaps systematically co-occurred, predominantly driven by the simultaneous absence of external validation, calibration assessment, and reproducibility or accessibility artifacts. The use of advanced algorithmic architectures (e.g., deep learning) and multimodal data integration did not independently reduce the reporting gap burden.

**Conclusion and relevance:**

In AI-based ASCVD risk prediction, reported algorithmic discrimination is a poor surrogate for clinical readiness. The literature features models with high discrimination but critical methodological deficits. These findings suggest that evaluation frameworks should consider incorporating calibration, external validation, and formal decision-analytic utility assessment alongside discrimination metrics.

## 1 Introduction

Cardiovascular disease (CVD), of which atherosclerotic cardiovascular disease (ASCVD) is a major component, remains the leading cause of global mortality and morbidity (1). Accurate risk stratification is the cornerstone of contemporary preventive cardiology and dictates the allocation of pharmacological therapies and lifestyle interventions. While traditional risk-estimating systems, such as the Pooled Cohort Equations (PCE) in the United States (2) and SCORE2 in Europe (3), are deeply embedded in clinical guidelines, they are inherently constrained by proportional hazards assumptions and a restricted set of conventional risk factors. More recently, the Predicting Risk of Cardiovascular Disease EVENTs (PREVENT) equations (4, 5) have been introduced to provide contemporary risk estimation with additional risk factors and competing-risk considerations, representing a meaningful advance in traditional risk assessment. To overcome these limitations, artificial intelligence (AI) and machine learning (ML) have been widely applied to develop novel ASCVD prediction models (6, 7). These computational approaches promise to improve risk classification by capturing complex, non-linear interactions across high-dimensional, multi-modal datasets, spanning electronic health records, circulating biomarkers, and advanced cardiovascular imaging.

Despite the rapid growth of AI-based predictive algorithms in the cardiovascular domain, the field confronts a substantial and persistent translation gap (8). Hundreds of published models report superior discrimination performance—routinely achieving areas under the receiver operating characteristic curve (AUC) or C-indices well above 0.80 or 0.90 (9). Nevertheless, evidence supporting the clinical adoption of these algorithms remains limited. The academic literature contains a rapidly expanding body of isolated, proof-of-concept models that may stall at the publication phase (10). This dynamic may create an appearance of rapid technological progress while the evidence base supporting real-world patient care or shared clinical decision-making remains underdeveloped. While several prior systematic reviews have cataloged AI-based cardiovascular prediction models (11, 12), they have predominantly focused on model performance comparisons or specific algorithmic families. The present study distinguishes itself by shifting the analytical unit from performance evaluation to a structured methodological audit, quantifying the prevalence, accumulation, and co-occurrence of translational-evidence gaps across the full spectrum of AI-based ASCVD prediction research.

This translational gap raises questions about the prevailing evaluation paradigm: the implicit assumption that algorithmic discrimination is an adequate proxy for clinical deployability. In practice, discrimination metrics merely rank individuals; they do not guarantee reliable absolute risk estimation (calibration), safety across diverse and unseen populations (external validation), or measurable improvements in decision thresholds (clinical utility) (13, 14). A clinically actionable model also requires a strictly defined temporal prediction horizon and a technologically feasible implementation pathway (15). In this study, deployability is operationally defined through these core methodological dimensions, and the Translational-Readiness Proxy represents our strict composite evaluation threshold. We posit that the absence of these core elements creates structured reporting and translational-evidence gaps that may impede clinical translation. Consequently, the entrenched reliance on AUC as the primary metric of success may mask severe underlying methodological deficiencies, leading to a potential discordance between reported model performance and translational readiness (16).

To investigate this phenomenon empirically, we conducted a systematic review combined with a structured methodological audit of AI-based ASCVD prediction models. By shifting the analytical unit from the conventional “performance-centric” assessment to a “methodological structure-centric” evaluation, this study characterizes the prevalence, accumulation, and structural co-occurrence of five critical methodological gaps (calibration, generalizability, clinical utility, temporal definition, and reproducibility/accessibility). Ultimately, we aim to examine the performance–deployability discordance hypothesis: evaluating whether high reported discrimination performance is associated with methodological completeness or whether structured reporting gaps represent potential barriers to the real-world translation of cardiovascular AI.

## 2 Methods

### 2.1 Study design and search strategy

We conducted a systematic review with a structured methodological audit, conducted in accordance with the PRISMA 2020 guidelines (17). Our primary objective was to evaluate how comprehensively published AI and ML models for ASCVD prediction report the evidence needed for clinical translation, specifically examining whether reported algorithmic discrimination is associated with prespecified reporting and translational-evidence gaps.

We searched five electronic databases (PubMed, Embase, Web of Science Core Collection, Scopus, and Cochrane CENTRAL) from 1 January 2020 to 27 March 2026 (Figure 1). The search strategy integrated three conceptual domains: ASCVD-related terminology, prognostic framing, and AI/ML methodology. Full search queries, tailored with database-specific syntax and Boolean operators, are provided in the Supplementary material to enable exact replication. See Supplementary Table S1 for the complete Boolean queries per database.

**Figure 1:**
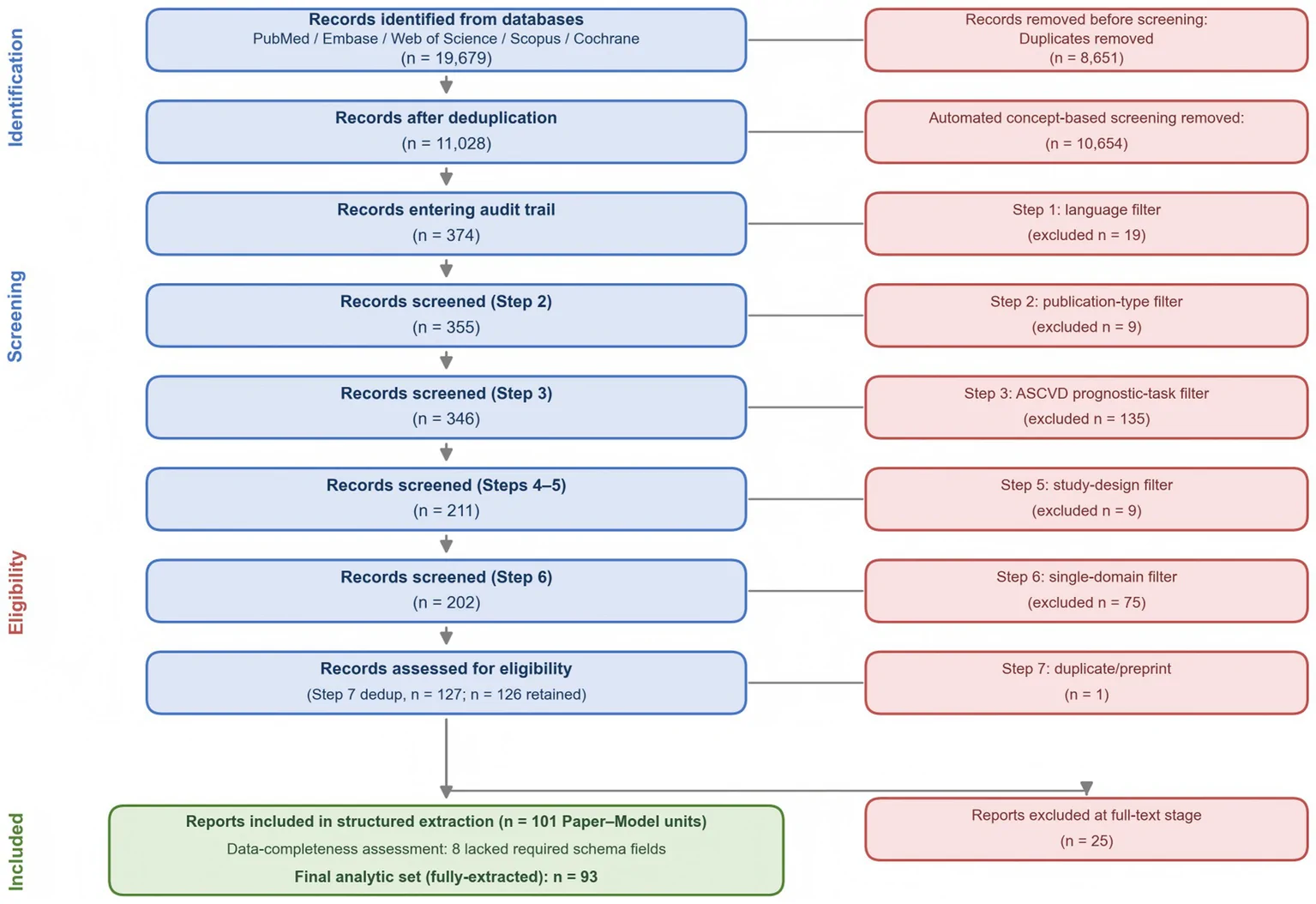
Study selection flow diagram according to PRISMA 2020. The PRISMA flow diagram illustrates the study selection process. Of the 19,679 records identified, 8,651 duplicates were removed, leaving 11,028 records for screening. Automated concept-based screening removed 10,654 non-relevant records, and 374 records entered the audit trail. Sequential eligibility filters excluded 19 records at the language step, 9 at the publication-type step, 135 at the ASCVD prognostic-task step, 9 at the study-design step, 75 at the single-domain step, and 1 duplicate/preprint, leaving 126 reports assessed for eligibility. After 25 reports were excluded at the full-text stage, 101 reports underwent structured extraction as primary paper–model units. A subset of 93 models with completely extractable schema fields was retained for predefined sensitivity analyses, as 8 of the 101 models lacked isolated schema fields. PRISMA, Preferred Reporting Items for Systematic Reviews and Meta-Analyses. The complete list of all 101 included studies with their model-level characteristics, discrimination metrics, gap counts, and translational-readiness classifications is provided in Supplementary Table S11.

### 2.2 PICOS framework and protocol registration

This review used the PICOS elements. Population (P): individuals at risk for ASCVD in whom risk prediction is clinically relevant; Intervention/Index model (I): AI/ML models developed or validated for ASCVD risk prediction; Comparator (C): traditional risk equations (e.g., PCE, SCORE2, Framingham) or other AI models, where reported; Outcomes (O): predictive discrimination (AUC/C-index), calibration, external validation, clinical utility, and translational-readiness indicators; Study design (S): peer-reviewed original studies reporting model development or validation. A formal *a priori* protocol was not registered on PROSPERO because the primary objective was a methodological audit rather than a pooled effect-size synthesis; however, all eligibility criteria, search queries, extraction schemas, and audit rules were prespecified prior to full-text screening and publicly archived in the open-access reproducibility package (see Supplementary material) to ensure complete transparency. The reproducibility package is timestamped for verification: the systematic search was finalized on 27 March 2026, and the prespecified extraction schema and audit rules were archived on 6 April 2026, before formal data analysis. Full-text screening was conducted between April and June 2026, confirming that all prespecified materials were archived prior to the start of full-text assessment.

### 2.3 Record processing and automated screening pipeline

Initial study selection used a custom Python-based text-mining pipeline (Python 3.12). Following automated deduplication via hierarchical matching of Digital Object Identifiers (DOIs) and PubMed IDs (PMIDs), the unique records underwent rule-based screening. The algorithm assessed predefined inclusion criteria using regular expression matching applied to normalized text, evaluating the co-occurrence of ASCVD endpoints, explicit algorithmic terminology, temporal definitions, and quantitative prognostic metrics.

The concept-based automated pipeline was retrospectively validated on a random sample of *n* = 200 records (seed = 20,260,812) using two rule variants (Reviewer A: ≥4/6 concept layers; Reviewer B: broadened ≥3/6 with expanded synonyms). Retrospective agreement rate and Cohen’s kappa are reported in Supplementary Table S7; the full protocol is in Supplementary material S2. The automated pipeline demonstrated high agreement with manual reviewer consensus, supporting its use as a reliable first-pass screening tool. To further minimize the risk of missing relevant studies, all records excluded by the automated pipeline were subsequently verified through manual inspection of a random sample, and no eligible studies were identified among the automatically excluded records in this verification subsample.

Records failing to satisfy all algorithmic criteria were excluded, yielding a candidate cohort for manual full-text evaluation. The entire screening script, including pattern definitions and execution logs, is open-sourced and provided in the Supplementary material (Concept-Based Screening.py) to guarantee full computational reproducibility.

### 2.4 Full-text eligibility, model selection, and data extraction

Two blinded investigators independently performed full-text review and data extraction using a standardized electronic case report form (eCRF). The eCRF was piloted on 15 randomly selected candidate studies before formal extraction. Discrepancies were resolved via consensus or adjudication by a third senior investigator. Studies were sequentially excluded if they lacked original prognostic modeling, evaluated non-ASCVD endpoints, used single-domain inputs without biological integration, or constituted non-primary literature.

For operational clarity, a “single-domain” input model was defined as drawing its predictor set exclusively from exactly ONE of the following categories: clinical/laboratory/history, imaging, biomarker/omics, functional/exercise, or ECG/signal without biological integration (e.g., using a calcium score or hs-troponin alone). Models categorized as “Clinical-only,” “Imaging-only,” or “Biomarker-only” in Table 1 were retained because they incorporated multiple within-domain predictor classes that satisfied the integration requirement (Supplementary Table S8). The complete 135-variable extraction schema and variable definitions are provided in Supplementary Table S2. For example, a ‘Clinical-only’ model remains eligible, as it typically integrates multiple predictor classes such as demographics, laboratory values, and medical history.

**Table 1:** Baseline characteristics, methodological GapBurden, and translational-readiness of included AI-based ASCVD prediction models (*N* = 101).

Characteristic	N	% (95% CI)
Data modality		
Clinical+Imaging	40	39.6
Clinical	23	22.8
Clinical+Biomarker	13	12.9
Multi-modal	9	8.9
Imaging	8	7.9
Biomarker	5	5.0
Imaging+Functional	3	3.0
Modeling approach		
Traditional ML	94	93.1
Multi-modal fusion	4	4.0
DL single-modality	3	3.0
Validation strategy^a^		
Internal validation	65	64.4
External validation	18	17.8
Cross-validation only	17	16.8
Temporal validation	1	1.0
Prediction task type		
Time-to-event	69	68.3
Binary fixed horizon	32	31.7
Discrimination performance^b^		
Median (IQR)	0.790 (0.734–0.833)	—
Range	0.590–0.965	—
Gap burden category		
Low (0–1 gap)	7	6.9
Moderate (2–3 gaps)	46	45.5
High (4–5 gaps)	48	47.5
Methodological gap prevalence^c^		
Calibration	59	58.4 (48.7–67.5)
Generalizability	83	82.2 (73.6–88.4)
Clinical utility	50	49.5 (40.0–59.1)
Temporal definition	40	39.6 (30.6–49.4)
Reproducibility/accessibility	91	90.1 (82.7–94.5)
Translational-readiness proxy met^d^	7	6.9

Data are presented as No. (%) unless otherwise indicated. ^a^For models using multiple validation strategies, the highest hierarchical tier of validation was recorded (External > Temporal > Internal/CV). ^b^Continuous discrimination metrics (AUC or C-index) were extractable from 98 of the 101 included models. ^c^Values in parentheses represent 95% confidence intervals calculated using the Wilson score method. ^d^Predefined composite threshold requiring GapCount ≤ 1, independent external validation, and formal clinical utility reporting. This is referred to throughout as the Translational-Readiness Proxy. AI, artificial intelligence; ASCVD, atherosclerotic cardiovascular disease; CI, confidence interval; CV, cross-validation; DL, deep learning; ML, machine learning.

Because reporting varied considerably, the primary unit of analysis was the “Paper–Model” unit. For publications reporting on the development or validation of multiple algorithms, a single primary model was selected based on a strict, predefined hierarchy: the model evaluated on the most rigorous dataset (external validation > temporal validation > internal test set) was prioritized. If multiple models shared the same validation tier, the model with the highest discrimination performance was selected to conservatively prevent underestimating the state-of-the-art in the field. Inter-rater reliability for the extraction of the methodological gaps was assessed using Cohen’s kappa (κ).

As illustrated in Figure 1, of the 19,679 initial records retrieved, 8,651 duplicates were removed, leaving 11,028 records for automated concept-based title/abstract screening. A total of 10,654 non-relevant records were excluded, and 374 records entered the formal audit trail. Sequential eligibility filters excluded 19 records at the language step, 9 at the publication-type step, 135 at the ASCVD prognostic-task step, 9 at the study-design step, 75 at the single-domain step, and 1 duplicate/preprint, leaving 126 reports assessed for eligibility. Of these, 25 reports lacked sufficient information for structured extraction and were excluded, and the remaining 101 studies advanced to structured data extraction (Figure 1).

### 2.5 Data-completeness assessment and analytical subsets

Of the 126 studies that entered the full-text stage, 25 could not supply sufficient information on model architecture, predictor set, or performance metrics to complete the 135-variable extraction schema and were excluded at the data-completeness gate; study-specific reasons are documented in the audit reconciliation file (PRISMA_Reconciliation.xlsx).

Consequently, 101 primary paper–model units were retained as the primary cohort for baseline characteristics and headline audit statistics. Within this cohort, 96 models provided complete paired data (discrimination metrics and gap counts) for correlation and scatterplot analyses. Additionally, eight studies lacked the isolated schema fields required for secondary sensitivity analyses, leaving an *n* = 93 fully extracted analytical set. All primary associations and stratified analyses were re-evaluated on this 93-model subset to confirm the robustness of the primary findings (Supplementary Table S5).

### 2.6 The structured methodological audit framework

Informed by the TRIPOD (13) and PROBAST (18) guidelines, we operationalized clinical deployability into five distinct methodological dimensions. This audit framework is an author-developed operationalization informed by TRIPOD reporting items and PROBAST bias domains; the resulting scores support study-level pattern evaluation and transparent re-scoring rather than serving as a formal PROBAST risk-of-bias rating. This structured audit also serves as the study-level risk-of-bias and applicability assessment for the present systematic review, with its five domains aligned conceptually with the participant, predictor, outcome, and analysis domains of PROBAST. Each dimension was coded as a binary variable (0 = gap absent, indicating methodological completeness; 1 = gap present, indicating a deficiency) based on predefined, verifiable thresholds. Since the initial development of this audit framework, updated reporting and assessment guidelines have been published, notably TRIPOD+AI (2024) and the forthcoming PROBAST+AI extension (19, 20). TRIPOD+AI provides updated reporting recommendations for prediction models using regression or machine-learning methods, while PROBAST+AI is expected to extend bias assessment to algorithmic fairness and implementation dimensions. Our five-domain framework was developed independently to capture the specific translational-evidence bottlenecks most relevant to clinical deployment rather than replicating comprehensive reporting checklists. The five domains align conceptually with key PROBAST domains (Participants, Predictors, Outcomes, Analysis) and TRIPOD reporting items, but are deliberately operationalized as binary, verifiable indicators to enable reproducible, field-level pattern detection across a methodologically heterogeneous corpus of 101 models. A detailed mapping of our five domains to TRIPOD+AI and PROBAST items is provided in Supplementary Table S4.

#### 2.6.1 Calibration assessment gap

Assigned (coded as 1) if the study failed to report any interpretable calibration measure such as a calibration plot, calibration slope, calibration-in-the-large or intercept, observed-to-expected ratio, or equivalent assessment. The Hosmer–Lemeshow test or Brier score alone was not considered sufficient evidence of calibration (21).

#### 2.6.2 Generalizability gap

Assigned (coded as 1) if the model was exclusively evaluated using internal data splitting (e.g., random train-test splits or k-fold cross-validation) without validation in a geographically, institutionally, or temporally distinct cohort.

#### 2.6.3 Decision-analytic utility evidence gap

Assigned (coded as 1) if the study did not conduct decision curve analysis (DCA) (14) or equivalent net benefit assessment at clinically relevant thresholds. To operationalize clinical utility, an actionable output requires a decision threshold or recommended clinical action; prototype demonstrators (web calculators or EHR add-ons) without prospective evaluation, Net Reclassification Improvement (NRI), or Integrated Discrimination Improvement (IDI) were treated as supplementary evidence but did not independently satisfy this criterion.

#### 2.6.4 Temporal definition gap

Assigned (coded as 1) if the prediction horizon was not explicitly defined in discrete time units (e.g., “10-year risk”) prior to modeling, or if the model relied on a variable observation window (classified as ‘Follow-upDuration’ reporting only median follow-up time) without time-dependent performance evaluation (Supplementary material S1).

#### 2.6.5 Reproducibility and accessibility artifact gap

Assigned (coded as 1) if the authors provided no accessible artifact permitting independent replication or execution [e.g., model coefficients or objects, input variable definitions, preprocessing specifications, algorithm details, public code repository, web calculator, Application Programming Interface (API), or deployable nomogram]. The absence of such artifacts does not necessarily indicate that clinical implementation is infeasible but limits independent verification. We note that this domain captures the availability of implementation-enabling artifacts (e.g., code, model objects, calculators) rather than the quality or completeness of the implementation itself. The term ‘Reproducibility/Accessibility’ is used to emphasize independent verifiability as the evaluation criterion, distinguishing it from assessments of active clinical deployment.

### 2.7 Composite audit metrics

We computed a GapCount for each model by summing the five binary variables (range, 0–5). Models were stratified into three GapBurden tiers: low (0–1 gap), moderate (2–3 gaps), and high (4–5 gaps). To establish a minimum evidence threshold, we also defined a prespecified translational-readiness proxy. A model was classified as meeting this proxy only if it simultaneously satisfied three criteria: GapCount ≤1, external validation, and reporting of decision-analytic utility metrics. This proxy establishes a pragmatic evidentiary baseline for potential translation and does not imply regulatory clearance, active clinical deployment, or proven patient benefit. For studies reporting multiple models on the same evaluation dataset, the author-designated primary model was retained for headline metrics, with secondary model estimates evaluated in clustered sensitivity analyses (Supplementary Table S5).

### 2.8 Statistical analysis

Statistical analyses evaluated the “methodological architecture” as the primary explanatory variable. Because continuous variables were non-normally distributed (Shapiro–Wilk test, Q–Q plots), we used non-parametric tests throughout.

Gap prevalence was estimated with Wilson score 95% confidence intervals (Figure 2). To test the performance–deployability discordance hypothesis (Figure 3), models lacking extractable continuous discrimination metrics were excluded pairwise. Given the mathematical differences between standard ROC-AUC, Harrell’s C-index, and time-dependent AUCs, discrimination metrics were synthesized descriptively rather than mathematically pooled via random-effects meta-analysis. As a prespecified sensitivity analysis, Spearman correlations were also computed separately for AUC-only models, C-index-only models, binary fixed-horizon models, and time-to-event survival models to assess whether the principal null finding was robust across metric types and prediction settings (Supplementary Table S10).

**Figure 2:**
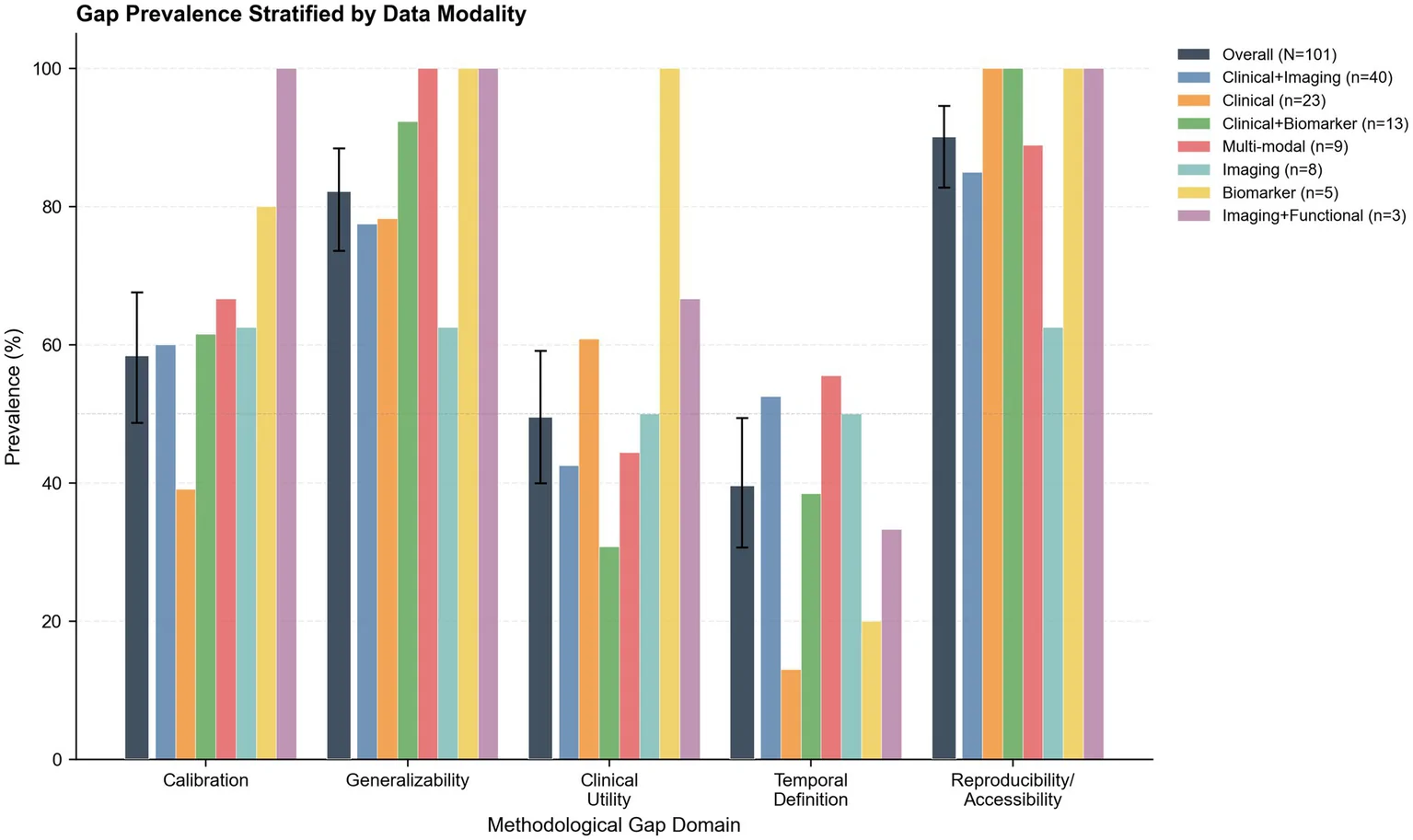
Prevalence of methodological gaps stratified by data modality. Prevalence of five predefined methodological gaps (calibration, generalizability, clinical utility, temporal definition, and reproducibility/accessibility) across 101 AI-based ASCVD prediction models, stratified by data modality. Bars represent the proportion of models with each gap present within each modality category. Error bars indicate 95% confidence intervals calculated using the Wilson score method. Overall prevalence estimates are shown alongside modality-specific distributions. Data modality categories include Clinical, Imaging, Clinical+Imaging, Clinical+Biomarker, Biomarker, Multi-modal, and Imaging+Functional. Gap definitions follow the structured methodological audit framework: calibration (absence of calibration assessment), generalizability (absence of external validation), clinical utility (absence of decision-analytic or comparative evaluation), temporal definition (unclear or undefined prediction horizon), and reproducibility/accessibility (absence of verifiable artifacts for independent replication).

**Figure 3:**
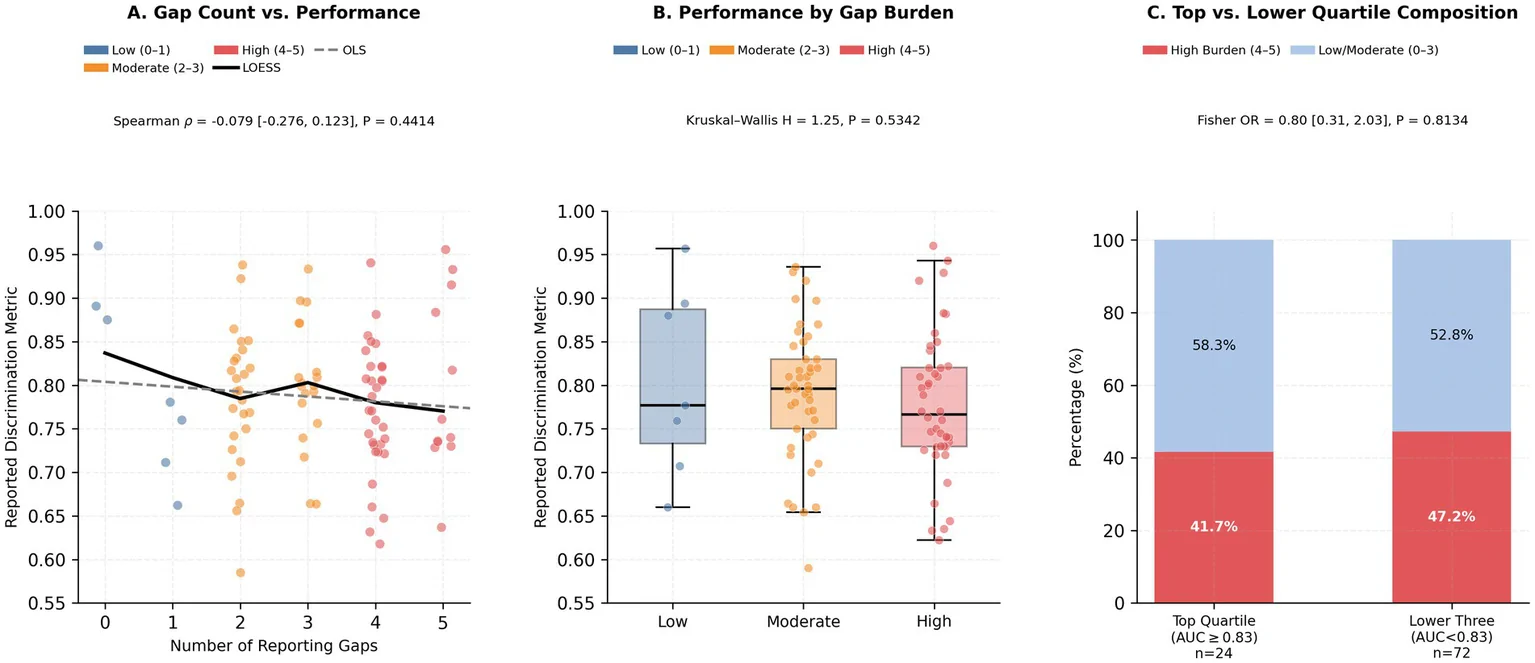
Discordance between reported discrimination and methodological completeness. Association between discrimination performance and cumulative methodological gap burden across AI-based ASCVD prediction models. **(A)** displays the relationship between GapCount (range, 0–5) and discrimination performance (AUC or C-index) among models with available performance data (*n* = 96), with locally estimated scatterplot smoothing (LOESS) and ordinary least squares (OLS) regression lines. **(B)** shows the distribution of discrimination performance stratified by GapBurden categories: low (0–1 gaps), moderate (2–3 gaps), and high (4–5 gaps). **(C)** compares the distribution of GapBurden between the top quartile of models (AUC ≥ 0.833, *n* = 24) and the remaining models (*n* = 72). GapCount represents the sum of five predefined methodological gaps (calibration, generalizability, clinical utility, temporal definition, and reproducibility/accessibility), each coded binary (1 = gap present, 0 = absent). GapBurden categories are defined as low (0–1), moderate (2–3), and high (4–5). In **(A)**, each point represents an individual model; dashed and solid lines denote OLS regression and LOESS smoothing, respectively; the Spearman correlation coefficient (ρ) and corresponding *p*-value are reported. In **(B)**, boxplots display the median and interquartile range, with whiskers representing 1.5 × IQR; group differences were assessed using the Kruskal–Wallis test. In **(C)**, bars represent proportions within each performance stratum; comparisons were performed using Fisher’s exact test, with odds ratios (OR) reported for High GapBurden. AUC, area under the receiver operating characteristic curve; OLS, ordinary least squares; LOESS, locally estimated scatterplot smoothing; OR, odds ratio; CI, confidence interval.

The relationship between GapCount and the primary discrimination metric (AUC/C-index) was evaluated using Spearman’s rank correlation (ρ) and ordinary least squares (OLS) regression, visualized via locally estimated scatterplot smoothing (LOESS). Discrimination distributions across GapBurden strata were compared using the Kruskal–Wallis H test. The prevalence of High GapBurden among top-quartile performers (AUC of 0.833 or higher) was compared with the lower three quartiles using the Fisher’s exact test. To examine the robustness of this null association, we performed sensitivity analyses stratified by discrimination metric type (AUC-only, *n* = 67; C-index-only, *n* = 31) and prediction setting (binary fixed-horizon, *n* = 32; time-to-event, *n* = 69). All subgroups confirmed the absence of a significant correlation between discrimination performance and methodological gap burden (Supplementary Table S10).

A 5 × 5 conditional probability matrix, P(A∣B), was computed for all gap pairs. This matrix was subjected to agglomerative hierarchical clustering (average linkage, Euclidean distance) to identify the underlying phenotypes of methodological failure.

Multivariable logistic regression identified potential correlates of High GapBurden and Translational-Readiness. Covariates—data modality, modeling approach, validation strategy, and log-transformed sample size—were selected *a priori* on clinical and methodological grounds. Variance inflation factors confirmed the absence of critical multicollinearity (all VIF < 5). Missing baseline covariate data (<5% across all variables) were handled using complete-case analysis. Model fit was evaluated using McFadden’s Pseudo-R^2^ and the Akaike Information Criterion (AIC).

Analyses used Python 3.12 with the pandas, scipy, statsmodels, and scikit-learn libraries. A fixed random seed ensured the reproducibility of clustering and smoothing. All tests were two-sided; a *p*-value of < 0.05 was considered significant. The primary analysis was the Spearman correlation between GapCount and discrimination performance, which was designated the confirmatory test. All other analyses—including the logistic regression, Kruskal–Wallis comparisons, conditional probability matrix, subgroup analyses, and sensitivity analyses—were considered exploratory and hypothesis-generating. Given the multiple exploratory comparisons performed, nominal *p*-values should be interpreted with caution, and findings from exploratory analyses require independent replication.

## 3 Results

### 3.1 Literature retrieval and baseline model characteristics

In total, 101 primary Paper–Model units met all eligibility criteria and were included in the final structured methodological audit. Table 2 details the operational definitions for the five methodological gap dimensions assessed in this study (calibration, generalizability, clinical utility, temporal definition, and reproducibility/accessibility). Inter-rater agreement for the five binary methodological gap variables was substantial to almost perfect (Cohen’s κ range: 0.81–0.95), indicating high consistency in audit coding. The domain-level results reported below jointly constitute the study-level risk-of-bias and applicability assessment for this systematic review.

**Table 2:** Operational definitions and clinical rationale for the structured methodological audit framework.

Gap dimension	Operationalization	Source field	Rule	Clinical rationale
Calibration	Calibration not reported	Calibration reported	Gap present (coded as 1) if the study did not report any interpretable calibration measure	Without calibration, predicted probabilities may be unreliable for clinical thresholds despite good discrimination.
Generalizability	No external validation	External validation	Gap present (coded as 1) if the model was not externally validated	Models validated only internally may overfit and fail to generalize to new populations or settings.
Clinical utility	Clinical utility not reported	clinical utility reported	Gap present (coded as 1) if the study did not report clinical utility assessment	Without DCA, NRI, or comparison to established scores, added clinical value is undemonstrated.
Temporal definition	Prediction horizon unclear/undefined	Window type	Gap present (coded as 1) if the prediction time window was recorded only as follow-up duration or was unclear	Ambiguous prediction windows prevent clinical application (clinicians need “risk within Y years”).
Reproducibility/accessibility	No publicly accessible reproducibility artifact (model coefficients, code repository, API, web calculator, or complete preprocessing specification)	Reproducibility artifact (derived from Implementability + Deployment readiness)	Gap present (coded as 1) if no artifact enabling independent replication was publicly available or accessible upon reasonable request	Absence of verifiable artifacts limits independent replication, external audit, and clinical adaptation of the model.

^a^The structural audit framework was theoretically informed by TRIPOD reporting items and PROBAST risk-of-bias domains. ^b^Each methodological dimension was strictly operationalized as a binary variable to ensure computational reproducibility. A score of 1 indicates a methodological deficiency (gap present), whereas a score of 0 indicates methodological completeness (gap absent). For evidence synthesis purposes, unreported methodological steps were strictly coded as unperformed (gap was coded as 1). This framework constitutes a structured methodological audit informed by TRIPOD and PROBAST domains and is not a formal PROBAST risk-of-bias rating. API, application programming interface; DCA, decision curve analysis; NR, not reported; NRI, net reclassification improvement; PROBAST, Prediction model Risk of Bias Assessment Tool; TRIPOD, transparent reporting of a multivariable prediction model for individual prognosis or diagnosis.

Baseline study and model characteristics are summarized in Table 1. Seven data modality groups were represented, with Clinical+Imaging (*n* = 40, 39.6%) and Clinical-only models (*n* = 23, 22.8%) being the most prevalent. The remaining models used Clinical+Biomarker (12.9%), Multi-modal (8.9%), Imaging-only (7.9%), Biomarker-only (5.0%), and Imaging+Functional (3.0%) inputs. Traditional machine learning predominated (93.1%, *n* = 94).

Most models relied on internal validation (64.4%, *n* = 65) or cross-validation (16.8%, *n* = 17). Only 17.8% (*n* = 18) of all evaluated models underwent independent external validation, and 1.0% (*n* = 1) used temporal validation. Among the 98 models (97.0%) with extractable continuous discrimination metrics, the median reported discrimination metric (AUC or C-index) was 0.790 (IQR, 0.734–0.833; range, 0.590–0.965). AUC and C-index were treated as approximate indices of discrimination but are not strictly interchangeable. Complete paired performance data for correlation analyses were available for 96 models.

### 3.2 Prevalence and accumulation of methodological gaps

Methodological gaps were pervasive across the AI-based ASCVD prediction literature (Figure 2). The most frequent deficiency was the Reproducibility/Accessibility gap, present in 90.1% of models (91/101; 95% CI, 82.7–94.5%), indicating an absence of accessible deployment pathways. Generalizability was the second most common gap, affecting 82.2% of models (83/101; 95% CI, 73.6–88.4%). Furthermore, 58.4% (59/101; 95% CI, 48.7–67.5%) exhibited a calibration gap (failure to report calibration), 49.5% (50/101; 95% CI, 40.0–59.1%) had a Clinical Utility gap, and 39.6% (40/101; 95% CI, 30.6–49.4%) demonstrated a Temporal Definition gap. Gap prevalence was broadly distributed across all data modality groups without any single technology demonstrating substantially lower deficiency rates.

Gaps accumulated within individual models rather than occurring as isolated omissions (Figure 4A). The distribution of the composite GapCount was right-skewed, with a mean of 3.20 and a median of 3. The modal category was a GapCount of 4 (32.7%, *n* = 33). Only three models (3.0%) were entirely free of gaps, and four (4.0%) possessed exactly one gap. Overall, 67.3% (68/101) of the models exhibited three or more concurrent gaps. Stratification by GapBurden found that approximately half of the cohort (47.5%, *n* = 48) fell into the high burden category (4–5 gaps), 45.5% (*n* = 46) into the moderate category (2–3 gaps), and only 6.9% (*n* = 7) fell into the low category (0–1 gap) (Figure 4B).

**Figure 4:**
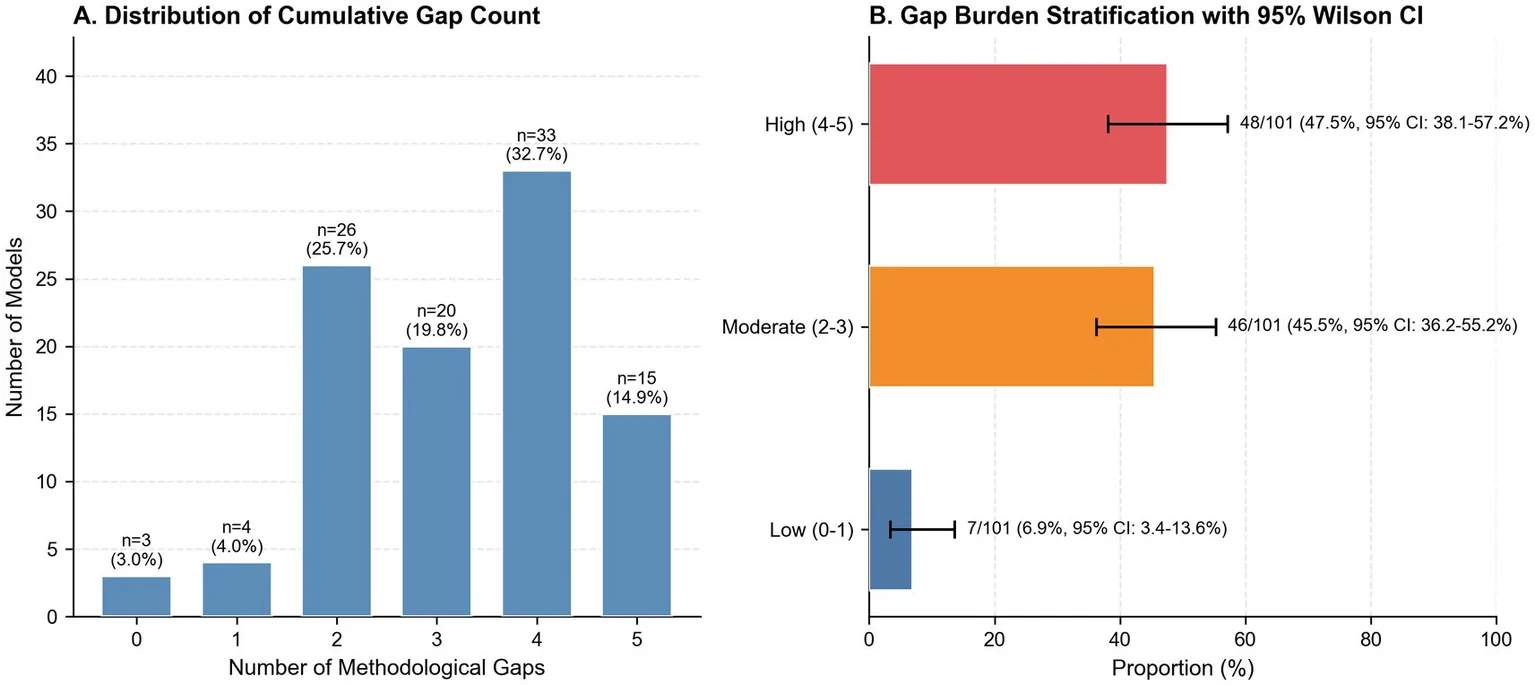
Distribution and stratification of cumulative methodological gap burden. Distribution of cumulative methodological gaps across 101 AI-based ASCVD prediction models. **(A)** shows the frequency distribution of GapCount (range, 0–5), defined as the total number of predefined methodological deficiencies present in each model. **(B)** presents the stratification of models into three GapBurden categories: low (0–1 gaps), moderate (2–3 gaps), and high (4–5 gaps), with corresponding proportions. GapCount was calculated as the sum of five binary methodological dimensions (calibration, generalizability, clinical utility, temporal definition, and reproducibility/accessibility), each coded 0 (no gap) or 1 (gap present). In **(A)**, bars represent the number of models within each GapCount category; numbers above bars indicate model counts, and proportions are provided in the text. In **(B)**, the horizontal bar chart illustrates the relative distribution of GapBurden categories. Color coding is consistent across panels: blue indicates low burden, orange indicates moderate burden, and red indicates high burden. GapCount, cumulative methodological gap count; GapBurden, gap burden category (low 0–1, moderate 2–3, high 4–5).

### 3.3 Structural co-occurrence patterns

Conditional probability analysis with hierarchical clustering showed that these gaps formed exploratory co-occurrence patterns (Figure 5). Reproducibility/accessibility gaps exhibited the highest conditional probabilities than with other dimensions; given the presence of a reproducibility/accessibility gap, the probability of concurrently manifesting generalizability and calibration gaps was markedly elevated. Hierarchical clustering suggested two exploratory co-occurrence groupings: an “evidence infrastructure” cluster comprising reproducibility/accessibility, generalizability, and calibration gaps, and an “interpretability infrastructure” cluster grouping clinical utility and temporal definition gaps.

**Figure 5:**
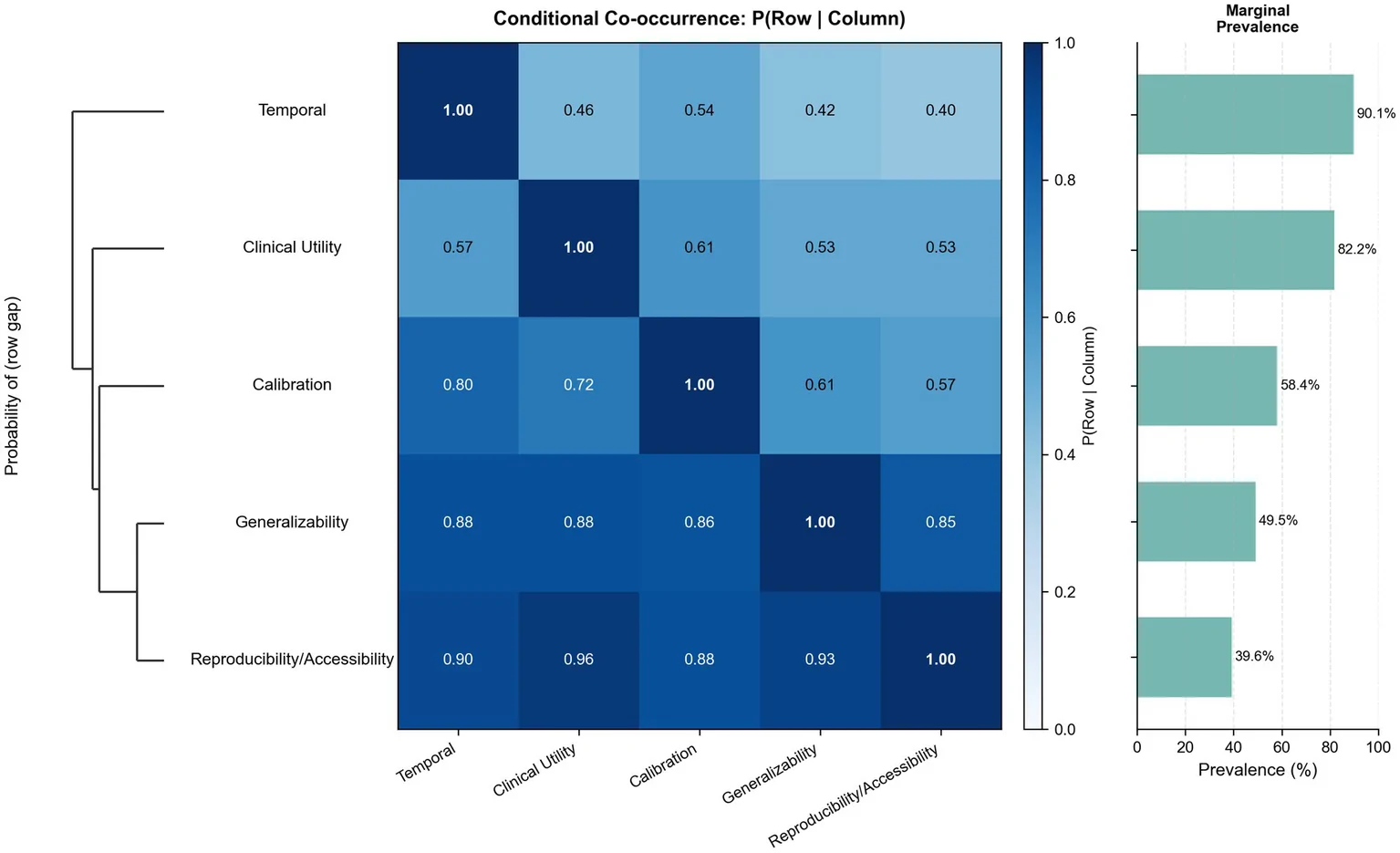
Conditional co-occurrence of methodological gaps and hierarchical clustering structure. Conditional probability matrix of pairwise methodological gap co-occurrence, defined as P(Row∣Column), across 101 AI-based ASCVD prediction models. Each cell represents the probability that a given gap (row) is present conditional on the presence of another gap (column). Color intensity corresponds to the magnitude of the conditional probability. Selected cells are annotated with numerical estimates, with diagonal elements indicating marginal prevalence. Agglomerative hierarchical clustering (average linkage, Euclidean distance) is applied to the row dimension to visualize similarity in co-occurrence patterns. Rows and columns correspond to the five predefined methodological gaps: reproducibility/accessibility, generalizability, clinical utility, calibration, and temporal definition. Conditional probabilities were computed from binary gap indicators (1 = gap present, 0 = absent). Values labeled “prev.” denote a marginal prevalence for the corresponding gap. The dendrogram (left panel) represents hierarchical clustering of gaps based on their conditional co-occurrence profiles. Warmer colors indicate higher conditional probabilities. P(A|B), conditional probability of gap A given gap B.

Temporal structure analysis (Figure 6A) showed that most models targeted medium- to long-term prediction horizons (e.g., 3–5 or 5–10 years), with short-term predictions (≤1 year) being rare. Trend analysis (Figure 6B) indicated that temporal definition gaps were disproportionately concentrated among models with higher cumulative GapBurden, reinforcing the structural nature of these deficiencies. The reported χ^2^ statistic (χ^2^ = 28.78, *p* < 0.001) was derived from a chi-square test to assess the association between temporal definition gap status and GapBurden strata.

**Figure 6:**
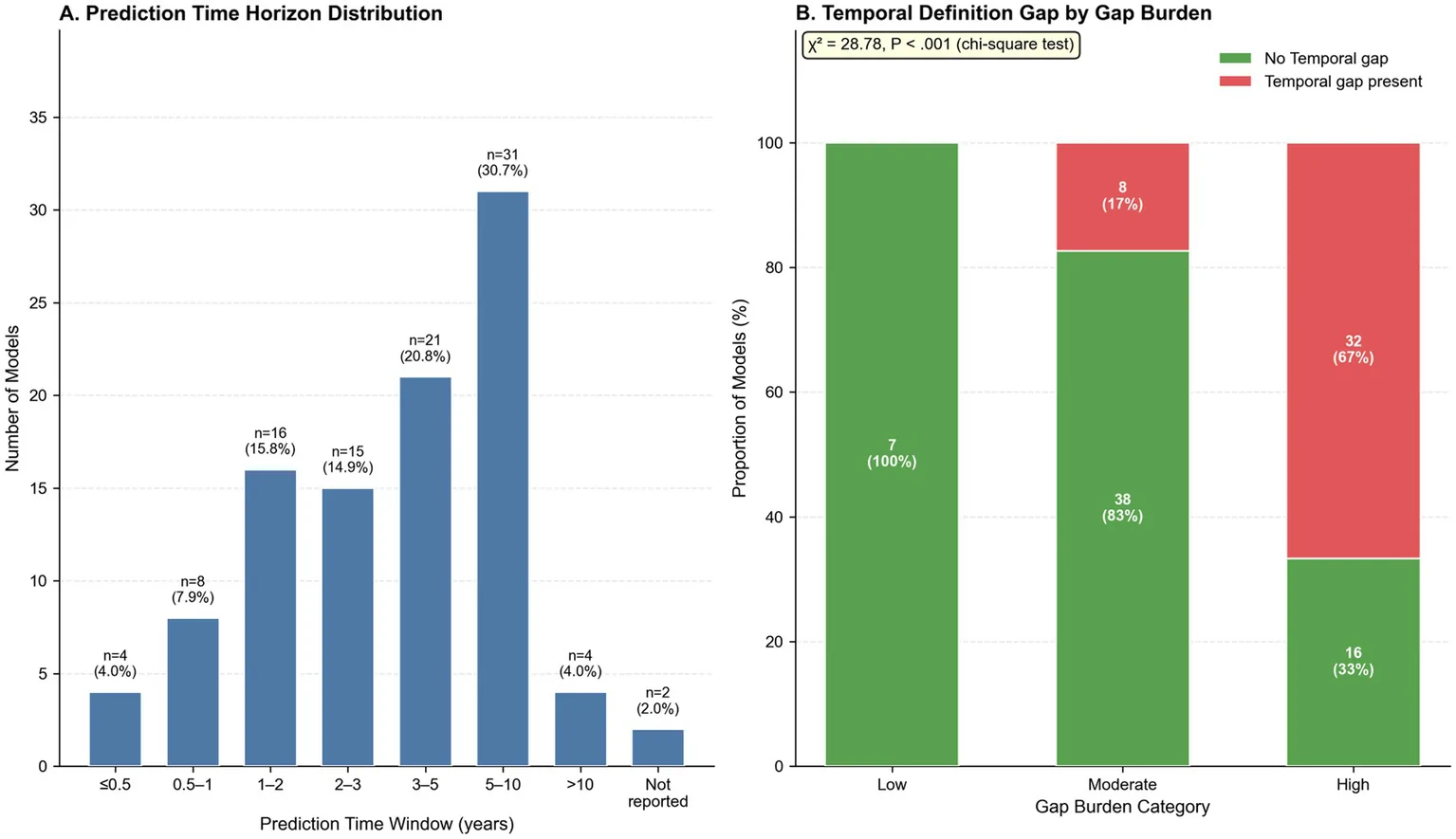
Prediction time horizon distribution and temporal definition gap across gap burden strata. Distribution of prediction time horizons and their association with temporal definition gaps among 101 AI-based ASCVD prediction models. **(A)** displays the frequency distribution of predefined prediction time windows. **(B)** shows the proportion of models with and without a temporal definition gap across GapBurden strata (low, moderate, high), with statistical comparison using the chi-square test. In **(A)**, bars represent the number of models within each prediction time window category, with percentages indicating proportions of the total sample (*N* = 101). Prediction horizons were extracted as reported and grouped into discrete intervals (≤0.5 year, 0.5–1 year, 1–2 years, 2–3 years, 3–5 years, 5–10 years, and >10 years). In **(B)**, stacked bars display the proportion of models with a Temporal Definition gap (red) and without a gap (green) within each GapBurden category. GapBurden was defined as low (0–1 gaps), moderate (2–3 gaps), and high (4–5 gaps). The reported χ^2^ statistic and corresponding *p*-value were derived from a chi-square test assessing the association between temporal definition gap status and GapBurden strata.

### 3.4 The performance–deployability discordance

Among 96 models with complete paired continuous discrimination metrics and gap counts, we did not detect evidence of an association between reported algorithmic discrimination and methodological completeness; however, this null finding should not be interpreted as evidence of equivalence (Figure 3). LOESS smoothing and OLS regression demonstrated a flat trajectory in discrimination performance across cumulative reporting gaps (Spearman ρ = −0.079, *p* = 0.441; OLS slope = −0.003) (Figure 3A).

Kruskal–Wallis testing did not detect evidence of a difference in AUC distributions across Low, Moderate, and High GapBurden strata (Figure 3B). Comparing the top-performing quartile (AUC ≥ 0.833, *n* = 24) against the remaining 75% (*n* = 72), there was no significant difference in the likelihood of High GapBurden (Fisher’s exact test OR = 0.80, 95% CI: 0.31–2.03, *p* = 0.813) (Figure 3C).

### 3.5 Determinants of gap burden and translational readiness

When applying the stringent Translational-Readiness Proxy (defined as GapCount ≤ 1, external validation present, and clinical utility reported), only 6.9% (7/101) of the evaluated models met the criteria (Figure 7A). All seven models meeting this proxy had undergone external validation; most models were classified as research-only (77.2%) per the translational-readiness classification (Figure 7B).

**Figure 7:**
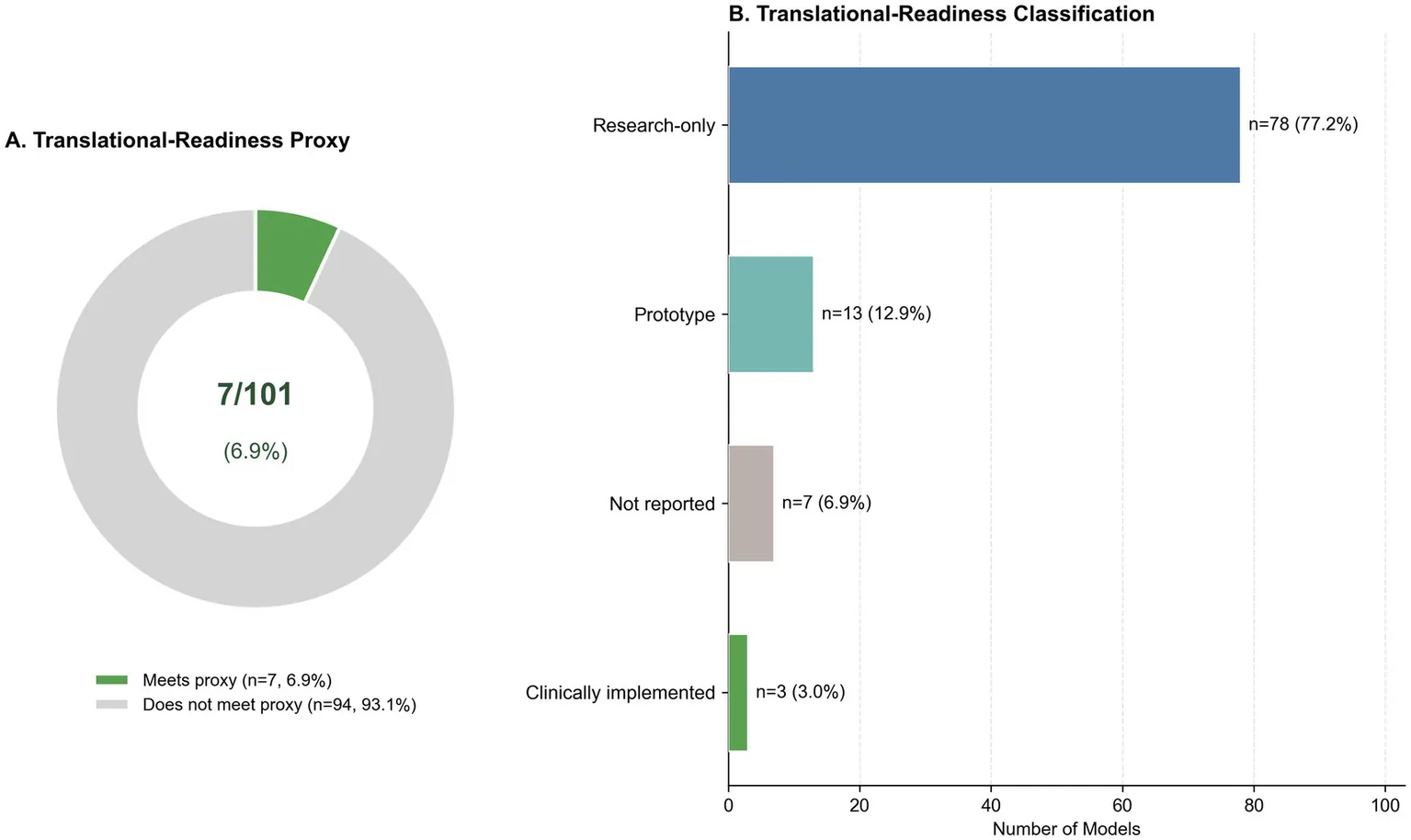
Translational-readiness proxy met and translational-readiness classification. Distribution of models meeting the predefined Translational-Readiness Proxy among 101 AI-based ASCVD prediction models. **(A)** presents the overall proportion meeting versus not meeting the proxy criteria (donut chart). **(B)** displays the translational-readiness classification of all models by reported development stage. The translational-readiness proxy was defined as meeting all of the following criteria: cumulative GapCount ≤1, presence of independent external validation, and reporting of clinical utility evaluation. In **(A)**, the donut chart displays the proportion of models meeting (7/101, 6.9%) or not meeting the proxy criteria. In **(B)**, horizontal bars represent the number of models classified by translational-readiness stage (research-only, prototype, not reported, clinically implemented), with percentages indicating proportions of the total sample (*N* = 101).

Subgroup analyses indicated that GapBurden distributions were largely independent of specific data modalities or modeling architectures; traditional machine learning models showed a 46% high GapBurden rate, whereas deep learning models showed a numerically higher rate (100%, *n* = 3); however, the deep learning subgroup was very small, and estimates were imprecise (Figure 8).

**Figure 8:**
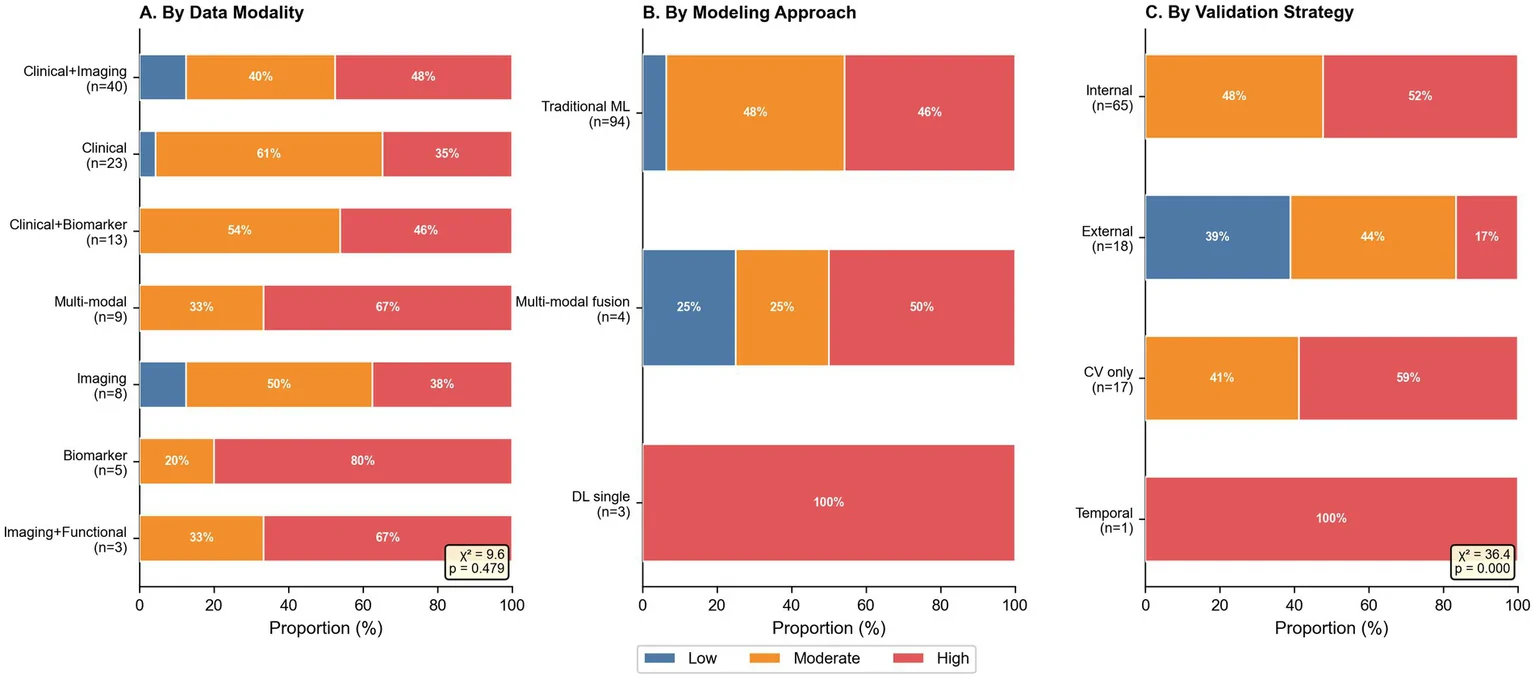
Distribution of methodological gap burden across model characteristics. Distribution of cumulative methodological gap burden stratified by key model characteristics among 101 AI-based ASCVD prediction models. **(A)** shows GapBurden distribution across data modality categories. **(B)** presents GapBurden distribution by modeling approach. **(C)** displays GapBurden distribution according to validation strategy, with statistical comparison across groups. GapBurden categories are defined as low (0–1 gaps), moderate (2–3 gaps), and high (4–5 gaps), based on the sum of five predefined methodological dimensions (calibration, generalizability, clinical utility, temporal definition, and reproducibility/accessibility). Stacked bars represent proportions within each subgroup, with colors indicating burden levels (blue = low, orange = moderate, red = high). Sample sizes for each subgroup are annotated. Data modality categories include Clinical, Imaging, Clinical+Imaging, Clinical+Biomarker, Biomarker, Multi-modal, and Imaging+Functional. Modeling approaches include traditional machine learning, deep learning (single-modality), and multi-modal fusion models. Validation strategies are categorized as internal (random split), cross-validation (CV only), external validation, and temporal validation. Group differences were assessed using chi-square tests, with corresponding χ^2^ statistics and *p*-values **(A,C)**; **(B)** was not formally tested because of sparse subgroups. Temporal validation included only 1 model; estimates are imprecise.

Multivariable logistic regression confirmed these descriptive findings (Table 3). In Model A [outcome: High GapBurden (4–5 gaps)], external validation emerged as the sole strong protective factor (OR = 0.08, 95% CI: 0.02–0.30, *p* < 0.001). Model complexity (e.g., deep learning approaches), sample size, and high-dimensional data modalities (e.g., multi-modal or imaging inputs) did not independently predict a reduction in methodological gaps. Because external validation constitutes one component of the GapCount outcome, the observed association between external validation and lower gap burden is partly structurally induced. To address this, a prespecified sensitivity analysis recalculated GapCount excluding the Generalizability component (yielding a 4-gap score) and re-estimated the logistic regression. In this sensitivity model, external validation remained inversely associated with gap burden, but the effect was substantially attenuated, and the 95% CI included null (OR = 0.20, 95% CI: 0.04–1.00, *p* = 0.049); the original estimate was therefore partly inflated by the outcome-definition overlap, and the attenuated association itself is fragile and should be regarded as hypothesis-generating. The primary conclusion that model complexity and data modality did not independently predict reduced gap burden was unchanged in the sensitivity analysis (Supplementary Table S9).

**Table 3:** Multivariable logistic regression analysis of factors associated with high gap burden (4–5 gaps).

Variable	Odds Ratio (OR)	95% CI	*P*-value
Intercept	2.37	0.77–7.29	0.131
Data modality (Ref: Clinical)			
Imaging-based	1.32	0.42–4.16	0.641
Multi-modal/biomarker	3.03	0.71–13.04	0.136
Modeling approach (Ref: Traditional ML)			
Deep learning-based	7.43	0.57–96.75	0.126
Validation strategy (Ref: Internal / CV only)			
External validation	0.08	0.02–0.30	< 0.001
Sample size (Ref: < median)			
Large sample (≥ median)	0.77	0.29–2.02	0.589

Model A goodness-of-fit statistics: Pseudo R^2^ = 0.198; Akaike Information Criterion (AIC) = 114.4; Total *N* = 101. Multivariable logistic regression evaluated factors associated with High GapBurden (presence of 4–5 gaps). *P*-values were derived from Wald tests. The translational-readiness proxy outcome was not modeled via regression due to sparse events (*n* = 7). AIC, Akaike information criterion; CI, confidence interval; CV, cross-validation; DL, deep learning; ML, machine learning; OR, odds ratio.

Among the seven models meeting the translational-readiness proxy, 100% (7/7) had undergone external validation, compared with 11.7% (11/94) in the non-meeting group. Due to sparse events in the proxy-meeting group (*n* = 7), multivariable regression was not performed for this outcome; the association between external validation and translational readiness is reported descriptively.

## 4 Discussion

### 4.1 Principal findings

This systematic review with structured methodological audit found that in AI-based ASCVD prediction, reported algorithmic discrimination was not associated with the prespecified composite of reporting and translational-evidence gaps. Models achieving higher AUC or C-index values were not systematically more methodologically complete, and only a marginal fraction met even a conservative proxy for translational readiness. These findings suggest that reported discrimination performance alone is insufficient as a surrogate for translational maturity. To contextualize this within a translational evidence hierarchy, we propose the following stages pathway: model development → internal validation → external validation → calibration and transportability assessment → decision-analytic utility evaluation → prospective clinical impact assessment → implementation and scalability → demonstrated patient benefit (22). Our audit shows that most of the included models have not progressed beyond the first two stages of this pathway, regardless of their reported discrimination performance. This framework aligns with the 2026 AHA scientific statement (23) on evaluating novel cardiovascular risk models, which emphasizes discrimination, calibration, incremental performance, clinical utility, scalability, generalizability, reproducibility, cost-effectiveness, and health equity as complementary evaluation dimensions.

#### 4.1.1 Limitations of discrimination metrics

The field continues to treat discrimination as the primary marker of predictive success. That convention is insufficient on its own to evaluate clinical usefulness. AUC and C-index quantify rank-ordering ability but do not indicate whether predicted probabilities are well-calibrated, whether accuracy holds outside the development setting, or whether model-guided decisions improve net outcomes (13, 14, 18, 21). In ASCVD prevention, clinical action depends on threshold-based risk estimation, not rank ordering alone. A model may perfectly separate higher-risk from lower-risk cohorts yet remain poorly calibrated, clinically unhelpful, or unusable in routine practice. Importantly, modest improvements in discrimination may still have clinical value when accompanied by improved calibration, treatment allocation, risk reclassification, or integration into preventive decision-making workflows.

#### 4.1.2 Systematic methodological deficits

Our results highlight a structural pattern in predictive modeling research: reporting gaps in methodological completeness were common regardless of reported discrimination performance. The widespread absence of calibration, external validation, utility evaluation, temporal framing, and implementation pathways suggests that most studies remain at the stage of technical demonstration. Similar bottlenecks have been described in clinical AI more broadly, where proof-of-concept performance often outpaces the evidence needed for safe deployment (8, 10, 15). The present study extends this critique to ASCVD risk prediction, showing that these translational gaps are not occasional oversights but a systemic structural deficit (24). Furthermore, evolving treatment paradigms, population characteristics, and healthcare delivery systems introduce temporal dataset drift that may progressively degrade model calibration and transportability over time if recalibration strategies are not incorporated.

#### 4.1.3 Structural clustering of gaps

The structural character of these deficiencies is important for understanding why translation stalls. Gaps did not appear in isolation but accumulated and clustered—most notably around external validity, calibration, and implementation. This clustering has practical implications. Investigators who do not validate beyond the development dataset are unlikely to report calibration or provide deployment pathways either. The pattern therefore reflects a study design orientation issue more than a series of discrete reporting failures. Much of the literature appears often focused on model development rather than the full pathway to clinical evaluation. Although reporting guidelines such as TRIPOD and risk-of-bias tools such as PROBAST were introduced precisely to mitigate these issues, our findings indicate that their core epidemiological principles remain incompletely assimilated into cardiovascular AI practice (13, 18).

#### 4.1.4 Methodological consideration of cox regression models

Approximately half of the included primary models used Cox proportional hazards regression. All Cox-based models retained in this audit satisfied at least one of the following criteria: (i) penalized feature selection using LASSO, Ridge, or Elastic-Net regularisation; (ii) tree-based or gradient-boosted variable-importance screening prior to Cox fitting; (iii) non-linear extensions such as Cox-nnet, DeepSurv, or spline-based Cox variants; or (iv) direct head-to-head comparison with a machine-learning survival model in the same cohort. Studies were further classified as (a) AI/ML-augmented Cox, (b) baseline Cox used as an ML comparator, or (c) traditional Cox; the audit’s main findings were unchanged in a sensitivity analysis excluding category (c) (Supplementary Table S6). The audit therefore focuses on the reporting and translational-evidence architecture of contemporary ASCVD prediction publications rather than on the mathematical family of the underlying estimator. Study-level Cox audit records (penalization, external validation, calibration, ML component, sample-size adequacy) are enumerated in Supplementary Table S3.

#### 4.1.5 Epidemiological vs. computational barriers

Equally notable is what did not predict gap burden. Neither data complexity nor model architecture consistently predicted methodological completeness. Richer data and more complex architectures did not protect against incomplete reporting. This argues against a purely technology-centric explanation for translational stagnation. The limiting barrier is not computational sophistication but the incomplete evidentiary pathway through which models are evaluated. Rigorous validation, transportability assessment, and decision-analytic evaluation remain prerequisites for useful prediction regardless of algorithm class modeling (14, 21, 25). We acknowledge that the logistic regression analysis is subject to a structural consideration: external validation is one component of the composite GapCount outcome and was simultaneously entered as an independent predictor. This creates a mathematical dependency that inflates the apparent protective effect of external validation in the original model. Our prespecified sensitivity analysis, which excluded the generalizability component from the outcome definition, demonstrated a substantially attenuated association that remains suggestive but imprecise, with the 95% CI including the null (OR = 0.20 vs. 0.08 in the original model). We therefore regard this finding as fragile and hypothesis-generating rather than confirmatory: it is directionally consistent with a genuine association between external validation and greater methodological completeness, although the estimate is imprecise; the magnitude of any independent association should be interpreted cautiously and requires confirmation in larger samples with non-overlapping outcome definitions. Future studies using independent outcome definitions and larger sample sizes are needed to confirm the independent contribution of external validation to translational readiness. Furthermore, the null association between discrimination and gap burden was consistent across all sensitivity analyses stratified by metric type and prediction setting (Supplementary Table S10), strengthening confidence in this principal finding.

#### 4.1.6 Translational implications

These findings have implications for authors, reviewers, and guideline bodies. First, reports introducing novel ASCVD prediction models should no longer be judged primarily by incremental gains in internal discrimination. Claims of clinical relevance should require calibration, independent validation, and utility evaluation. While decision curve analysis is not the only option, some demonstration that model outputs inform clinical action beyond standard care is needed (14). Second, implementation feasibility must be treated as a core component of the model evaluation rather than an optional addendum. Importantly, deployability, implementation, and proven clinical benefit should be viewed as distinct translational stages requiring progressively higher levels of evidentiary support. An algorithm with no reproducibility or accessibility artifacts may have limited independent verifiability, regardless of its statistical performance. However, non-reporting does not confirm that implementation is infeasible. Third, future systematic reviews and clinical guideline appraisals must explicitly separate predictive performance from translational readiness, as our data confirm these are not interchangeable constructs. These clinical implications should be interpreted within the broader context of contemporary cardiovascular risk assessment: AI-based models must demonstrate not only discrimination superiority but also calibration accuracy, transportability across populations, decision-analytic value, and equitable performance before they can be recommended for clinical implementation over established tools such as the PREVENT equations (4).

#### 4.1.7 Future directions

The next phase of cardiovascular AI research must pivot away from the repetitive derivation of *de novo* algorithms on isolated datasets. In a rapidly expanding field, the incremental translational value of publishing additional high-AUC, internally validated prediction models without accompanying translational evidence may be limited. Substantive progress will require an emphasis on transportability testing, algorithmic recalibration across diverse populations, head-to-head benchmarking against established risk equations, and prospective evaluation of model-supported decision-making (3, 8, 15). Emerging approaches such as multimodal foundation models, federated learning, and adaptive recalibration frameworks may partially address current limitations in transportability and scalability, although these strategies remain incompletely validated in preventive cardiology. Because ASCVD prevention is already anchored by validated guideline-endorsed tools (e.g., Pooled Cohort Equations, SCORE2), the evidentiary burden of proof for replacing them with AI models should be correspondingly high. Accordingly, near-term clinical integration may be more realistically achieved through augmentation or recalibration of existing guideline-based frameworks rather than wholesale replacement of established risk equations. Critically, the evaluation of cardiovascular AI models must also incorporate dimensions beyond traditional performance metrics. Although algorithmic fairness assessment was outside the scope of the present five-domain audit, health equity and algorithmic fairness deserve explicit attention in future evaluation efforts: AI-based ASCVD prediction models may perform differentially across sex, age, ethnicity, socioeconomic status, healthcare setting, and geographic region. A model with excellent overall discrimination but poor subgroup calibration or systematic bias in underrepresented populations may be clinically unsafe. The forthcoming PROBAST+AI framework is expected to emphasize fairness and algorithmic bias assessment, which our current binary audit does not capture. Furthermore, as contemporary cardiovascular prevention moves toward tools such as the PREVENT equations (4), the evidentiary standard for AI-based models must encompass not only discrimination but also calibration, transportability, clinical utility, cost-effectiveness, and equitable performance across diverse populations.

### 4.2 Strengths and limitations

This study has several limitations. First, this audit relied on published reports and their Supplementary material. If essential methodological steps were performed but omitted from the study, they were coded as absent; ‘not reported’ should not be equated with ‘not performed’. However, in the context of evidence synthesis, non-reported methodology cannot be appraised, reproduced, or trusted for clinical translation; enforcing this standard is why reporting guidelines exist (13). Second, to maximize reproducibility and minimize subjective grading bias, the five methodological dimensions were operationalized as strict binary variables. This binary audit framework necessarily compresses nuanced methodological quality into dichotomous indicators, meaning partial or mathematically flawed attempts at validation or utility assessment were not differentiated from complete absence. Third, the translational-readiness proxy was intentionally pragmatic; it establishes a minimum evidentiary threshold for plausible clinical translation and should not be conflated with true clinical deployment, regulatory clearance, or proven patient benefit. Fourth, aspects not captured by our proxy—including fairness, subgroup performance, model updating, data drift, interoperability, privacy, cost-effectiveness, and clinical impact studies—represent additional dimensions relevant to clinical translation. Fifth, the exclusion of non-English literature may limit the capture of regionally specific models. Sixth, by restricting our analysis to a single primary model per paper (Paper–Model unit), within-study heterogeneity among secondary algorithms was not retained; the selection of the highest-performing model per paper may introduce upward performance bias. Seventh, heterogeneity in outcomes, populations, prediction horizons, event rates, and validation approaches limits direct comparability of discrimination metrics across studies, and publication bias may inflate observed performance. Eighth, although the automated concept-based screening pipeline is fully open-source, and its rule-based criteria are documented in the Supplementary material, a formal sensitivity-specificity validation against a manually reviewed random sample of automatically excluded records was not performed for the current synthesis. Ninth, the risk-of-bias appraisal was implemented via our prespecified structured methodological audit. Tenth, our structured audit framework was developed prior to the publication of TRIPOD+AI (2024) and the anticipated PROBAST+AI extension (19, 20); consequently, we did not perform a formal item-by-item assessment using these updated frameworks. While our five domains align conceptually with key elements of both TRIPOD+AI and PROBAST, a comprehensive re-evaluation using these newer tools would be a valuable direction for future research. Finally, the multiple exploratory comparisons performed in this study increase the risk of type I error; the primary confirmatory analysis was the Spearman correlation, and all other findings should be considered hypothesis-generating pending independent replication.

These limitations are offset by several notable strengths, including a prespecified methodological audit framework, fully reproducible screening protocols, explicit operational definitions, and an analytical design that prioritized structural methodology over conventional performance metrics. This framework allowed the field to be evaluated on parameters relevant to clinical translation, rather than the metrics most frequently reported.

## 5 Conclusion

In summary, AI-based ASCVD prediction research exhibits a pattern in which reported discrimination performance frequently exceeds the completeness of supporting translational evidence. Reported discrimination was frequently moderate to high; evidence meeting a prespecified translational-readiness proxy was rare. The principal finding is not that predictive performance is unimportant, but that it is insufficient as a sole indicator of translational readiness. Evaluating algorithms with the same epidemiological rigor applied to other clinical interventions—including calibration, external validation, temporal clarity, decision-analytic utility, and reproducibility—may help advance the field beyond models that are statistically promising yet insufficiently supported for clinical translation. Ultimately, the future success of cardiovascular AI will likely depend less on algorithmic novelty and more on whether these models can show robust, reproducible, and clinically actionable benefit across diverse real-world populations.

## Data Availability

The raw data supporting the conclusions of this article will be made available by the authors, without undue reservation.
